# Knowledge and Awareness of Primary Teeth and Their Importance among Parents in Bengaluru City, India

**DOI:** 10.5005/jp-journals-10005-1334

**Published:** 2016-04-22

**Authors:** Jyothsna Vittoba Setty, Ila Srinivasan

**Affiliations:** 1 Professor, Department of Pedodontics and Preventive Dentistry Mathrushri Ramabai Ambedkar Dental College and Hospital Bengaluru, Karnataka, India; 2 Professor and Head, Department of Pedodontics and Preventive Dentistry Mathrushri Ramabai Ambedkar Dental College and Hospital Bengaluru, Karnataka, India

**Keywords:** Awareness, Knowledge, Parents, Primary teeth.

## Abstract

**Introduction:** Often people responsible for the oral care of children feel or believe that since primary teeth will eventually shed, it is not worthwhile to spend time/money on providing good oral health to children. Parents are the ones who take care of their children and make decisions for them. Hence, they should have knowledge about primary teeth, their health and caring in order to build confidence in their children through tiny teeth.

**Aim:** To assess the knowledge of primary teeth and their importance among parents with children below 12 years.

**Materials and methods:** A total of 1,000 questionnaires containing questions written both in English and in the local language (Kannada) were prepared for data collection and were personally distributed to parents visiting dental clinics for their children’s dental treatment.

**Statistical analysis:** Both descriptive statistics and Chi-square test were used.

**Results:** Complaints related to dental caries constituted 82% of children visiting dental clinics among children in Bengaluru city. Only 39% of respondents were aware of all functions of primary teeth.

**Conclusion:** The present study revealed that the parents of Bengaluru city had superficial or partial knowledge of primary teeth and that there is a need to improve this awareness.

**How to cite this article:** Setty JV, Srinivasan I. Knowledge and Awareness of Primary Teeth and Their Importance among Parents in Bengaluru City, India. Int J Clin Pediatr Dent 2016;9(1):56-61.

## INTRODUCTION

Primary teeth are the valuable assets of a child. In children, milk teeth/primary teeth play a vital role for eating, phonetics, esthetics and also as a space maintainer for permanent teeth. Often problems in milk teeth in the form of pain and swelling can cause distress to the child, leading to inability to chew or speak properly or even may affect the appearance of a child.

Young children’s dental environment is complex as parental knowledge, attitudes and beliefs affect the child’s oral health.^1,2^ As parents are the primary caregivers of their children they should have knowledge about the primary teeth, its health and caring in order to build confidence in their children.^3^ Parents are decision makers for their children. Sarnat et al^4^ reported that at the age of 5-6 years, the more positive the mother’s attitude toward dental health the better is the child’s oral hygiene. Therefore, it is important to examine the attitudes and also the knowledge of the parents, as these may affect their behavior toward their child’s oral health.

There has been a significant decline in the prevalence of dental caries in children in most of the industrialized countries on account of a conscientious effort on their part to promote oral health care of children. Children from low-income and disadvantaged families have been found to have high caries prevalence and poor oral health.^5^ In developing countries like India, there is limited documented research on parental awareness of primary teeth. So, the present study was undertaken to assess the knowledge, attitude and perceptions of parents of primary teeth in Bengaluru city, India.

## AIMS AND OBJECTIVES

- To assess the knowledge of primary teeth and their importance among parents with children below 12 years of age.
- To compare the influence of socioeconomic status on the knowledge, awareness and importance of primary teeth.

## MATERIALS AND METHODS

The study was conducted among parents of Bengaluru city, Karnataka, India. Prior approval for the study was obtained from the Institutional Ethical Committee, Mathrushri Ramabai Ambedkar Dental College and Hospital, Bengaluru. All parents of children aged up to 12 years who reported to the Department of Pedodon-tics and Preventive dentistry of Mathrushri Ramabai Ambedkar Dental College were invited to participate in the study. Voluntariness and strict confidentiality were assured; 1,000 questionnaires both in English and in the local language (Kannada) were personally distributed for data collection. Assistance was offered for those who desired help in understanding the questions.

The demographic details were collected from the parents, such as name, age, sex, educational qualification, address, monthly income, child’s age, number of children and the reason for visit to dental clinic. The responders were then asked to indicate the most appropriate correct answer from the given list of options in order to assess their knowledge, awareness and perception regarding importance of primary teeth.

The questionnaire assessed the parental knowledge and awareness about primary teeth, their location, number, functions, shedding and effects on permanent teeth. Further assessments of parents’ attitude toward treatment of decayed, traumatized or infected primary teeth and their willingness to comply with the treatment options for such teeth and also beliefs or taboos associated with extractions were made.

All over the world, social scientists have considered occupation as the most important determinant of the level of social standing of an individual in society. In India, Prasad’s classification of 1961, further modified in 1968 and 1970, is based on per capita income. Prasad’s classification has been used in most Indian studies and has been found to be effective in its task. The income limits emphasize only the need for updating this classification with time. Realizing this need, Kumar^6^ linked Prasad’s classification with the All India Consumer Price Index, as both of them shared the same base year of 1961. Thus, using the above method, the recent update of Prasad’s classification was used in our study.^7^ We considered classes I and II as high socioeconomic groups, class III as middle and classes IV and V as low socioeconomic groups.^8^

A total of 1,000 questionnaires were completed by the participants; 100 of them were excluded because they were either incomplete or someone other than the parent had completed the questionnaire or more than one option in the answers was ticked.

Collected data were tabulated and subjected for statistical analysis using Statistical Package for Social Sciences (SPSS) version 13.0. Frequency distribution which includes number and percentage was calculated. Chi-square analysis was used for comparison between different socioeconomic groups. The level of significance was set at p < 0.05.

## RESULTS

It was observed that mothers (58%) accompanied their children more than fathers (42%) for dental treatment (Table 1). Caries-related conditions, such as, pain/food impaction/sensitivity constituted 82% of reasons for the visit to dental clinic.

Results of the questionnaire are tabulated in Table 2.

The answers to questions regarding what parents pursue milk teeth/primary teeth showed ignorance among almost half of the participating parents (questions 1, 2 and 4 in Table 2).

**Table 1:** **Table 1:** Gender distribution among parents accompanying children for dental treatment

*Gender*		*n*		*Percentage*	
Male		324		36	
Female		576		64	
Total		900		100	

**Table 2:** **Table 2:** Responses to the questions by parents

*Question*		*Options*		*Responses in numbers (n)*		*Percentage*	
Q1		What are milk teeth/primary teeth/deciduous teeth?					
		Teeth which are present in the children drinking milk		234		26	
		Present in all children		144		16	
		First set of teeth which will be replaced by permanent teeth		468		52	
		None of these		54		6	
Q2		How many milk teeth/primary teeth are present totally?					
		All front teeth		189		21	
		All teeth in the mouth of 4-year-old children		459		51	
		Don’t know		180		20	
		All upper teeth		72		8	
Q4		How many teeth in the mouth of 3-year-old are primary?					
		50%		225		25	
		25%		234		26	
		None		90		10	
		All		351		39	
Q7		Total no. of primary teeth present					
		8		180		20	
		12		162		18	
		18		135		15	
		20		270		30	
		4		153		17	
Q3		Do you think all primary teeth will shed?					
		Yes		549		61	
		No		153		17	
		Only front teeth		189		21	
		Only back teeth		9		1	
Q5		By what age do you think all primary teeth will be replaced by permanent teeth?					
		4 years		108		12	
		6 years		189		21	
		12 years		477		53	
		18 years		126		14	
Q6		Do you think all the permanent teeth erupt by replacing their respective milk tooth?					
		Yes		423		47	
		No		153		17	
		Some of them		324		36	
Q8		Primary teeth help in:					
		Chewing		225		25	
		Appearance of child		54		6	
		Speech		18		2	
		Maintains the space for permanent teeth/guides the eruption of permanent teeth		63		7	
		I and ii		45		5	
		i, ii and iii		126		14	
		i, ii and iv		18		2	
		All of the above		351		39	
Q9		Do you think it is important to treat a decayed milk tooth?					
		Yes		774		86	
		No		826		14	
Q10		If a primary tooth is infected					
		It is important to save infected primary teeth if possible		684		76	
		It is unnecessary, since anyway tooth is going to fall		216		24	
Q11		If an infected primary teeth in your child’s mouth require extensive treatment probably requiring a few visits to the dental office and some expenditure					
		You will agree for treatment		540		60	
		You will not agree for treatment		360		40	
Q11		Reasons					
		Time		105		29	
		Economically difficult/expenditure		104		29	
		Unnecessary to spend time and money for a tooth which is anyway going to shed		151		41	
Q12		If an infected primary tooth require extraction which is the only possible treatment option					
		You will agree for extraction		666		74	
		You will not agree for extraction		234		26	
Q12		Reasons					
		Eyes will get affected		63		27	
		Brain will get affected		28		12	
		As the tooth will shed there is no need for extraction		63		27	
		Will cause pain/trauma in child		52		23	
		Expenditure		28		12	

Only 30% of parents were aware of total number of primary teeth present (question 7 in Table 2).

Knowledge regarding shedding of primary teeth and eruption of permanent teeth was not clear at least among half of the parents who participated in the study (questions 3, 5 and 6 in Table 2).

Among the respondents, only 39% of the parents were aware of all the functions of primary teeth (question 8 in Table 2).

**Graph 1:**
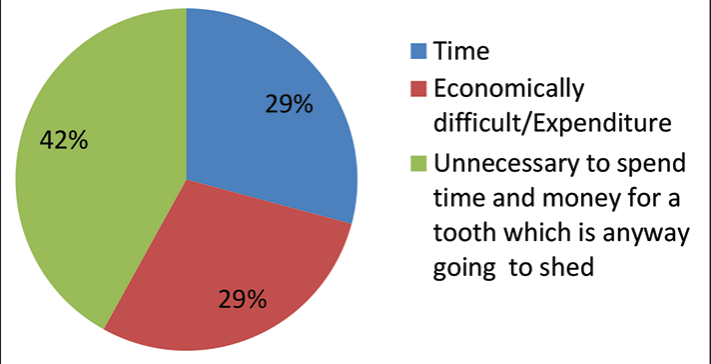
Reasons not willing for treatment

When asked about the importance of treating a decayed or infected primary tooth, majority of the parents (86 and 76%, questions 9 and 10 of Table 2) felt it is important to treat such teeth, although about 40% of them were not ready to spend time and money for treatment since they felt it is unnecessary as these teeth will shed (question 11 - Reasons; Table 2, Graph 1).

**Graph 2:**
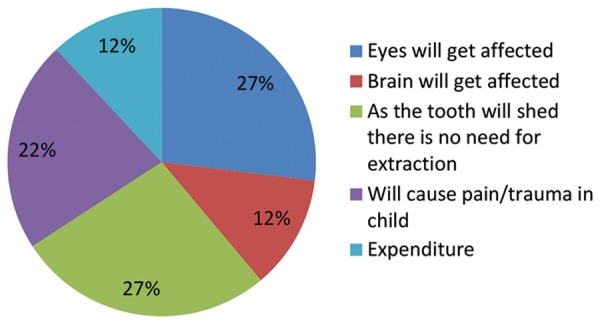
Reasons not willing for extraction

Willingness to comply with extraction as the only option left to treat the infected tooth was agreed by majority of them (74%) and only about 26% were not willing. This unwillingness was due to varied reasons like taboos associated with extraction, misconceptions like eyes and brain of the child may be affected, expenditure, procedures that might cause pain and trauma to the child or simply because primary tooth will anyway shed (question 12 - Reasons; Table 2, Graph 2).

The results of the questionnaire when compared between different socioeconomic groups showed no statistical significance (Table 3). The knowledge of primary teeth was relatively less among low socioeconomic groups as compared with middle and high socioeconomic groups. Their willingness to comply with different options for treatment was also less, probably because of their socioeconomic status.

**Table 3:** **Table 3:** Responses according to socioeconomic status

				*Class I* *(n = 270)*				*Class II* *(n = 216)*				*Class III* *(n = 233)*				*Class IV* *(n = 9)*							
*Question*		*Options*		*n*		*%*		*n*		%		*n*		%		*n*		%		*X^2^*		*p-value*	
Q1		Teeth which are present in the children drinking milk		72		27		36		17		81		24		5		56		12.642		0.179	
		Present in all children		49		23		9		4		63		19		1		11					
		First set of teeth which will be replaced by		108		40		153		71		180		54		3		33					
		permanent teeth																					
		None of these		27		10		18		8		9		3		0		0					
Q2		All front teeth		54		20		63		29		63		19		1		11		18.083		0.034	
		All teeth in the mouth of 4-year-old		180		67		99		46		153		46		3		33					
		Do not know		18		7		54		25		63		19		5		56					
		All upper teeth		18		7		0		0		54		16		0		0					
Q3		Yes		153		57		135		63		207		62		6		67		2.875		0.969	
		No		54		20		36		17		54		16		1		11					
		Only front teeth		54		20		45		21		72		22		2		22					
		Only back teeth		9		3		0		0		0		0		0		0					
Q4		50%		81		30		36		17		99		30		1		11		5.247		0.812	
		25%		981		30		54		25		72		22		3		33					
		None		9		3		36		17		36		11		1		11					
		All		99		37		90		42		126		38		4		44					
Q5		4 years		45		17		9		4		36		11		2		22		10.809		0.289	
		6 years		27		10		36		17		90		27		4		44					
		12 years		162		60		126		58		162		49		3		33					
		18 years		36		13		45		21		45		14		0		0					
Q6		Yes		153		57		108		50		126		38		4		44		8.003		0.238	
		No		54		20		36		17		36		11		3		33					
		Some of them		63		23		72		33		171		51		2		22					
Q7		8		81		30		18		8		72		22		1		11		10.812		0.545	
		12		54		20		35		21		54		16		1		11					
		18		45		17		36		17		36		11		2		22					
		20		81		30		63		29		108		32		2		22					
		24		9		3		54		25		63		19		3		33					
Q8		Chewing		72		27		45		21		90		27		18		22		17.856		0.658	
		Appearance of child		0		0		27		13		18		5		9		11					
		Speech		0		0		0		0		9		3		9		11					
		Maintains the space for permanent teeth		18		7		18		8		27		8		0		0					
		I and ii		32		13		0		0		9		3		0		0					
		i, ii and iii		32		13		27		13		54		16		9		11					
		i, ii and iv		9		3		0		0		9		3		0		0					
		All of the above		99		37		99		46		117		35		36		44					
Q9		Yes		234		87		207		96		261		78		72		89		3.786		0.286	
		No		36		13		9		4		72		22		9		11					
Q10		It is important to save infected primary teeth if possible		243		90		135		63		252		76		54		67		6.054		0.109	
		It is unnecessary, since anyway tooth is going to fall		27		10		81		38		81		24		27		33					
Q11		You will agree for treatment		243		90		198		92		243		73		63		78		5.130		0.163	
		You will not agree for treatment		27		10		18		8		90		27		18		22					
Q12		You will agree for extraction		198		73		189		88		207		62		72		89		6.012		0.111	
		You will not agree for extraction		72		27		27		13		126		38		9		11					

## DISCUSSION

Maintaining healthy primary teeth is essential to a child’s overall oral and general development.^9^ Parents and family members are considered the primary source for knowledge about child rearing and health habits for children, which undoubtedly have a long-term influence in determining a child’s oral health status.^10^ They are considered the key persons in achieving the best oral health outcomes and assuring well-being for children.

Frequently in pediatric dental practice we find parents ignorant about the primary tooth, its function and importance. They often question the necessity of treatment to save and maintain the milk tooth in function.

There is no good reason for leaving primary teeth decayed and untreated in a child’s mouth. No other branch of medicine would willingly leave disease untreated.^11^

Untreated carious primary tooth can give rise to different complications, such as pain, oral infection, problems in eating and sleeping, malnutrition and alterations in growth and development^12-15^ and probably early loss of teeth, which might lead to short-term effects like problems in eating and speaking and long-term effects like malalignment of permanent teeth and increased risk of malocclusion later on.^16^

In the present study, 82% of parents visited the dental clinic only after their child had complaints of untreated carious teeth; 39% of parents were aware of all the functions of primary teeth. The reason for poor knowledge among parents and low value about primary teeth might be due to cultural-based opinions or the fact that these are temporary teeth and they will shed and be replaced by a new set of secondary teeth. Some authors have reported that certain cultures place little value on primary teeth and that caries and early loss of the primary dentition is an accepted occurrence.^17^

A qualitative study of caregivers in Saipan found that the low value attributed to baby teeth was an obstacle to developing effective preventive program.^18^ In another qualitative study, Finnish caregivers of preschool children gave less importance to primary teeth when compared with general health.^19^

Conversely a Canadian study indicated that parents who believed baby teeth were important had children with significantly lower caries rates than those who believed otherwise.^20^ Thus, parental knowledge of primary teeth appears to have a direct effect on the oral health of the child.

## CONCLUSION

The present study revealed that the parents of Bengaluru city had superficial or partial knowledge and awareness of primary teeth and importance. There is a need to cultivate and reinforce positive attitude among parents and substantially raise their dental awareness through child dental health-oriented programs with active parental involvement. Such awareness programs should be developed for parents imparting knowledge about primary teeth, their function and preventive primary care of these teeth. To achieve this, young and prospective parents should be directed by the medical professionals, obstetricians, gynecologists and pediatricians to seek professional oral health counseling.
